# Elaboration of an instrument to evaluate the recognition of Brazilian melodies in children^☆^

**DOI:** 10.1016/j.bjorl.2018.05.011

**Published:** 2018-06-30

**Authors:** Maria Fernanda Capoani Garcia Mondelli, Ivan dos Santos José, Maria Renata José, Natália Barreto Frederigue Lopes

**Affiliations:** a Universidade de São Paulo (USP), Faculdade de Odontologia de Bauru, Departamento de Fonoaudiologia, Bauru, SP, Brazil; b Universidade de São Paulo (USP), Faculdade de Odontologia de Bauru, Programa de Pós-Graduação em Fonoaudiologia, Bauru, SP, Brazil

**Keywords:** Hearing, Hearing aids, Music, Child, Audição, Aparelhos auditivos, Música, Criança

## Abstract

**Introduction:**

There is evidence pointing to the importance of the evaluation of musical perception through objective and subjective instruments. In Brazil, there is a shortage of instruments that evaluates musical perception.

**Objective:**

To develop an instrument to evaluate the recognition of traditional Brazilian melodies and investigate the performance of children with typical hearing.

**Methods:**

The study was carried out after approval of the research ethics committee (1.198.607). The instrument was developed in software format with website access, using the languages PHP 5.5.12, Javascript, Cascade style sheets and “HTML5”; database “MYSQL 5.6.17” on the “Apache 2.4.9” server. Fifteen melodies of Brazilian folk songs were recorded in piano synthesized timbre, with 12 seconds per melody reproduction and four second intervals between them. A total of 155 schooled children, aged eight to 11 years, of both sexes, with typical hearing participated in the study. The test was performed in a silent room with sound stimuli amplified by a sound box at 65 dBNA, positioned at 0 azimuth, and at one meter from the participant, the notebook was used for children to play with on the screen on the title and illustration of the melody they recognized they were listening to. The responses were recorded on their own database.

**Results:**

The instrument titled “Evaluation of recognition of traditional melodies in children” can be run on various devices (computers, notebooks, tablets, mobile phones) and operating systems (Windows, Macintosh, Android, Linux). Access: http://192.185.216.17/ivan/home/login.php by login and password. The most easily recognized melody was “Cai, cai balão” (89%) and the least recognized was “Capelinha de melão” (25.2%). The average time to perform the test was 3′15″.

**Conclusion:**

The development and application of the software proved effective for the studied population. This instrument may contribute to the improvement of protocols for the evaluation of musical perception in children with hearing aid and/or cochlear implants users.

## Resumo

**Introdução:**

Há evidências que apontam para a importância da avaliação da percepção musical através de instrumentos objetivos e subjetivos. No Brasil, há escassez de instrumentos que avaliem a percepção musical.

**Objetivo:**

Desenvolver um instrumento para avaliar o reconhecimento de melodias tradicionais brasileiras e investigar o desempenho de crianças com audição típica.

**Método:**

O estudo foi desenvolvido após aprovação do comitê de ética em pesquisa (1.198.607). O instrumento foi desenvolvido em formato de software com acesso ao *site*, com as linguagens de programação PHP 5.5.12, Javascript, Cascade Style Sheets e HTML5 banco de dados MYSQL 5.6.17 no servidor Apache 2.4.9. Quinze melodias de canções folclóricas brasileiras foram gravadas com timbre sintetizado em piano, com 12 segundos de reprodução da melodia e quatro segundos de intervalo entre elas. Participaram do estudo 155 crianças, alfabetizadas, entre oito e 11 anos, de ambos os sexos, com audição típica. O teste foi feito em sala silenciosa com estímulo sonoro amplificado por uma caixa sonora a 65 dB NA, posicionada a 0 azimute e a um metro do participante e o notebook foi usado para que as crianças brincassem na tela com o título e a ilustração da melodia que eles reconheceram que ouviam. As respostas foram registradas em seu próprio banco de dados.

**Resultados:**

O instrumento intitulado “Avaliação do reconhecimento de melodias tradicionais em crianças” pode ser executado em vários dispositivos (computadores, notebooks, tablets, telefones celulares) e sistemas operacionais (Windows, Macintosh, Android, Linux). Acesse: http://192.185.216.17/ivan/home/login.php através de *login* e senha. A melodia mais facilmente reconhecida foi “Cai cai balão” (89%) e a menos reconhecida foi “Capelinha de melão” (25,2%). O tempo médio para fazer o teste foi de 3′15″.

**Conclusão:**

O desenvolvimento e aplicação do *software* se mostraram eficazes para a população estudada. Esse instrumento pode contribuir para o aprimoramento de protocolos de avaliação da percepção musical em crianças usuárias de próteses auditivas e/ou usuárias de implante coclear.

## Introduction

Sensory deprivation has lasting repercussions on brain development and behavioral outcomes.^1^, ^2^ In children with hearing loss, deprivation is negatively correlated with neural development,^2^ as well as perceptual, linguistic and cognitive abilities.^1^, ^3^, ^4^, ^5^ As the development and organization of cortical auditory pathways critically depends on sensory experience.^6^, ^7^ the restoration of auditory function with amplification devices is insufficient for children to hear correctly. Deaf children should learn to interpret auditory signals to create meaningful representations of sound and listening strategies.^8^

Access to speech perception is possible through the digital technology of sound amplification devices such as Hearing Aids (HA) and Cochlear Implant (CI). In Brazil, the acquisition of these resources can be performed in Hearing Health Services accredited by the Unified Health System. The basic function of these devices is to amplify the sound to maximize the speech spectrum available to the user, to improve speech audibility. One of the intended effects is to improve the development of speech and language.^9^

The link between language and music skills, and their overlapping processes, is of great interest. Music and language have common characteristics: both systems are composed of discrete elements (phonemes and notes), organized in temporal and hierarchical structures (words and chords), depend on the auditory processing of complex acoustic elements, and transmit rich meanings.^10^ Studies have shown a great overlap in the brain regions involved in the processing of music and language at cortical levels^11^, ^12^ and subcortical.^13^ These similarities, both in processes and in brain networks, can subjugate the effects of transferring from one domain to another in the normal population. Thus, training with one type of material (e.g., music) should improve its efficiency to process other types of stimuli, such as language.^14^, ^15^

However, the perception of music is unsatisfactory for users of sound amplification devices; music plays a fundamental role in the establishment of communicative abilities, since it is a powerful tool for transmitting emotion, identifying emotional clues and is an important part of human social and communication development.^16^

Music comprises numerous harmonics that vary in frequency over a wide range; musical melodies, even with a single instrument, are composed of a series of complex tones.^17^ Our perception of music is therefore influenced by the way the auditory system encodes and retains acoustic information.^18^

One of the salient features of sound relevant to music is tom.^19^ Besides the tone or the melody, the music depends on the rhythm. Behavioral studies demonstrate that rhythm and tone can be perceived separately,^20^ but that they also interact^21^ in creating a musical perception.

Currently, CI users and, to a certain extent, HA users, struggle with complex auditory perceptual tasks, particularly those requiring perceptual tuning^22^ and melodic contour.^23^ According to a recent study^24^ a standardized and accurate assessment of musical perception skills would offer new opportunities to investigate nonverbal auditory abilities such as timbre, rhythm, and melodic contour.

Several tests were designed for the musical perception test in CI users, including batteries such as the Primary Measures in Musical Audience, Music Excerpt Recognition Test, and Percussion Drum and Musical Assessment of Iowa.^25^, ^26^, ^27^ Many of these tests, while providing important information on various aspects of musical perception, require hours to complete, require the assistance of trained personnel and would be difficult to administer in a typical clinical environment. Studies at various institutions use different melodies in their recognition tasks, and the same melody can be presented in varying ways, from the use of different instruments and pitch registers to the use of simple melodic lines and actual recordings. Many of these recordings contain rhythmic clues that can contribute to melody recognition.^28^, ^29^ Researchers^30^ demonstrated that CI users performed significantly better on familiar melody recognition tasks when rhythmic cues were available. The lack of standardization in the test of musical perception also impedes the ability to compare the results of patients in different institutions.

The audiology practice guide^31^ states that assessments of amplification devices should be performed to verify the child's performance in auditory tasks with the use of them by means of speech-in-noise test and measurements with probe microphone, however in relation to music, there is no Brazilian test that evaluates the musical recognition of HA/CI users.

The hypothesis of the study is that this application can be used to perform the evaluation of the amplification devices, being an instrument of easy application.

The objective of the study was the development and validation of an instrument to evaluate the recognition of melodies in school children with normal hearing.

## Methods

The research initiated after approval of the Ethics Committee in Research (n° 46839315.7.0000.5417). According to the ethical precepts of research with human beings, the participants and those responsible were clarified about the fundamentals, objectives and procedures of the research, as well as about the benefits and absence of health risk and about the confidentiality related to the data obtained.

The transversal, descriptive and observational research was developed in two stages: creation and validation of the instrument.

### Development

The instrument was developed by a team composed of programmer, design and musician, after being given the specifications by the research team. We used PHP 5.5.12, Javascript, Cascade Style Sheets (CCS) and HTML5; database MYSQL 5.6.17, on the Apache 2.4.9 server. The computer program is authorized for academic and/or research purposes only, and may be used on different devices such as computer, notebook, tablets and mobile phones.

The melodies were recorded with synthesized piano tone, standardized with varying tempos according to each song, similar intensity, tone according to the score and reproduction of 12 s each and pause of four seconds between them.

The form of response to the stimulus of the melodies was by touching the screen, the test was organized so that the child performs a previous training to understand the activity. The melodies are performed in a random way, randomized by the program itself, amplified by an Alto Truesonic TS 115A sound box with intensity of 65 dBNA.

The recognition of the music depends on specific factors of the culture,^32^ in this way, the melodies considered most popular in Brazil were selected: “Atirei o pau no gato; Bate o sino; Boi da cara preta; Brilha, brilha estrelinha; Cai, cai balão; Capelinha de melão; Caranguejo; Escravos de Jó; Marcha, soldado; Nana nenê; Noite feliz; Ó ciranda, ó cirandinha; O cravo; Parabéns a você e Sambalelê”.

To identify the melodies, a screen was created with the names associated with figures that refer to the title.

After completion, the test itself generates a results sheet, where it is possible to check the correct, incorrect and unanswered alternatives, as well as the duration of the test application. The system stores the information.

### Instrument validation

Participants in the study of children's music were invited to participate in the study, children with normal hearing, who attended the Municipal Coexistence Centers and participated in children's music classes at this place. The data collection took place in the centers of coexistence during the school hours.

The children who participated met the following inclusion criteria:

Attend the municipal convention center in the evening period;
Signature of the free and informed consent form by the parents and endorsement by the children;
Meatoscopy without indication of presence of foreign body and/or excess of cerúmen;
Hearing within normality standards after screening (AAA).^33^

In the auditory screening, it was considered as a “pass/fail” criterion, and the child would pass if it responded to at least two of the three tones emitted^33^ at 20 dBNA for the frequencies of 1000, 2000 and 4000 Hz and 30 dBNA for 500 Hz in both ears.

The room where the procedures were performed was the school's quietest the noise level was monitored throughout the collection by means of instantaneous readings performed through the NoiSee program, installed on the iPad and maintained between 28 and 49 dB NPS. No noise was recorded that exceeded the values allowed by ANSI-S 3.1-1991. During the recreation periods, the collection did not occur, due to environmental noise.

After the consent of those responsible, the procedures were performed, all on the same day, in the following order:

Meatoscopy: to evaluate the conditions of the external auditory canal and tympanic membrane. If there was any obstruction that could interfere with the results, the child was referred to the otorhinolaryngologist for evaluation and conduct.

Auditory screening: performed using the Pediatric Audiometer (PA5) – Interacoustics with specific headset, the 0.50 kHz, 1 kHz, 2 kHz and 4 kHz thresholds were investigated for both ears separately.

Tympanometry: performed using the Titan – Interacoustics equipment, aiming to verify the tympano – ossicular and middle ear conditions.

Evaluation of the recognition of melodies: the child remained seated, positioned at 0° azimuth one meter from the sound box and was instructed to touch the screen when he recognized the melody he was listening to. The data referring to the number of correct answers, errors and absence of answers; reaction time for each response; total time of evaluation and graph of evaluation were obtained by the bank of answers of the test itself.

The tests used for statistical analysis were: Chi-square test, for variables, music, sex and age; Analysis of Variance and Tukey for the variables reaction time, gender and age. Quantitative variables were represented by mean, median, standard deviation and minimum and maximum values. In all tests, the level of rejection of the adopted null hypothesis was 5% (*p* < 0.05).

## Results

A total of 155 children participated in the study, 49% female and 51% male.

**Figure 1:**
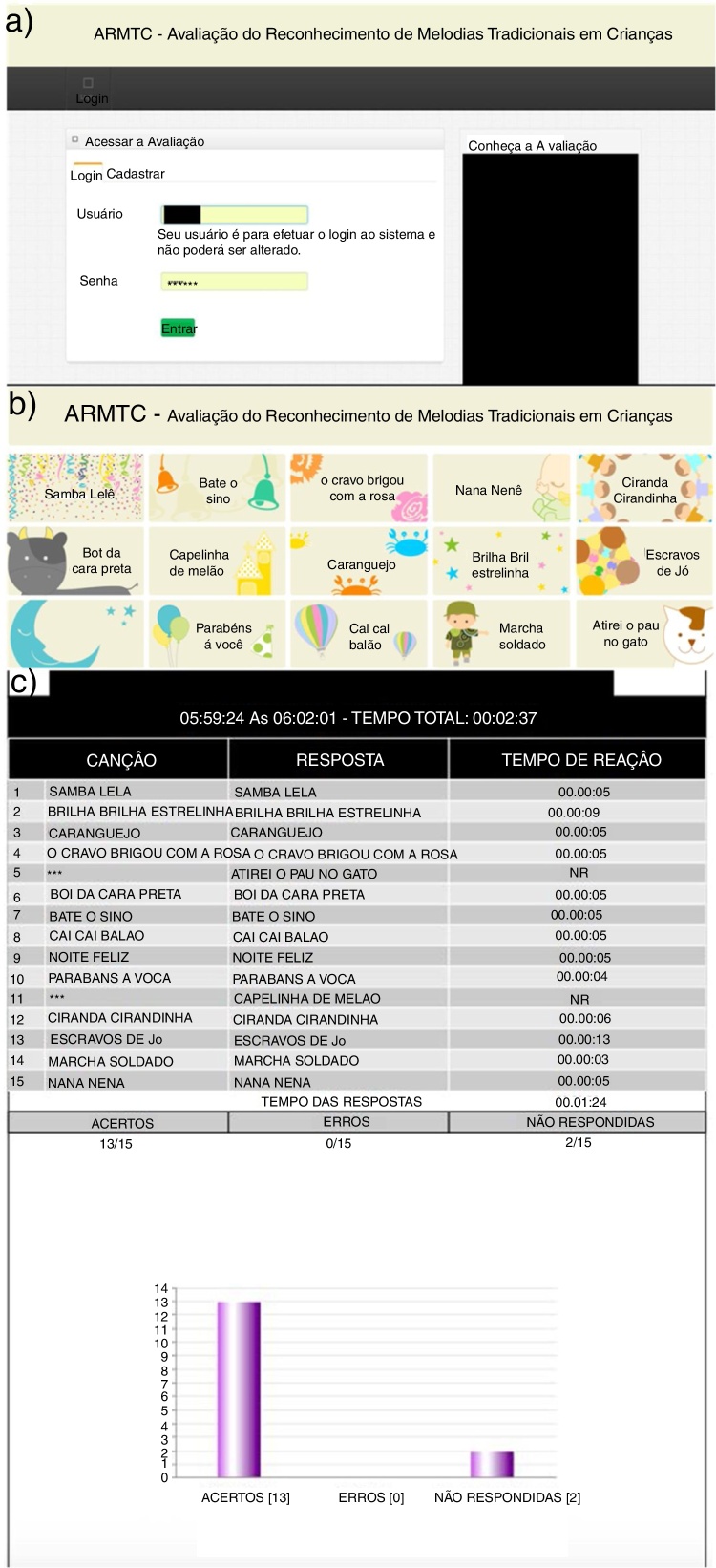
Instrument start screen (A), Evaluation screen (B) and Response screen (C).

The distribution by age group is shown in Table 1. According to the proposed objective, the instrument “Evaluation of the Recognition of Traditional Melodies in Children” was developed. Fig. 1(A–C) illustrates the software access screen, the evaluation screen composed of the images corresponding to the melodies and the results screen, respectively. The instrument application can be accessed: http://srv60.teste.website/∼ivan/home/login.php, with login and password.

**Table 1:** Distribution of the number of children according to age.

Age	*n*	%
8	38	24.5
9	43	27.7
10	39	25.2
11	35	22.6
Total	155	100

*n*, number.

The most easily recognized melodie was “Cai, cai balão” (89%) and the least recognized was “Capelinha de melão” (25.2%). The frequency distribution of errors and correctness for the total sampled children is shown in Table 2.

**Table 2:** Distribution of the frequency of errors and correctness in each song in the total sampled children.

Melodies	Right answers *n* (%)	Wrong answers *n* (%)
M1 – *Atirei o pau no gato*	90 (58.1)	65 (41.9)
M2 – *Bate o sino*	102 (65.8)	53 (34.2)
M3 – *Boi da cara preta*	123 (79.4)	32 (20.6)
M4 – *Brilha, brilha estrelinha*	72 (46.5)	83 (53.5)
M5 – *Cai, cai balão*	138 (89.0)	17 (11.0)
M6 – *Capelinha de melão*	39 (25.2)	116 (74.8)
M7 – *Caranguejo*	123 (79.4)	32 (20.6)
M8 – *Escravos de Jó*	122 (78.7)	33 (21.3)
M9 – *Marcha soldado*	109 (70.3)	46 (29.7)
M10 – *Nana nenê*	83 (53.5)	72 (46.5)
M11 – *Noite feliz*	83 (53.5)	72 (46.5)
M12 – *Ó ciranda, ó cirandinha*	67 (43.2)	88 (56.8)
M13 – *O cravo*	122 (78.7)	33 (21.3)
M14 – *Parabéns a você*	110 (71.0)	45 (29.0)
M15 – *Sambalelê*	99 (63.9)	56 (36.1)

*n*, number; M, melody.

According to the percentage of recognition, the melodies were thus ordered (highest to lowest): “Caranguejo”, “Boi da cara preta”, “O cravo”, “Escravos de Jó”, “Parabéns a você”, “Marcha soldado”, “Bate o sino”, “Sambalelê”, “Atirei o pau no gato”, “Nana nenê”, “Noite feliz”, “Brilha, brilha estrelinha” e “Ciranda, cirandinha”.

Table 3 shows the distribution and correlation of errors and correctness in each song according to age.

**Table 3:** Distribution and correlation of errors and correctness in each song according to age.

	8 years old		9 years old		10 years old		11 years old		*p*
	RA, *n* (%)	WA, *n* (%)	RA, *n* (%)	WA, *n* (%)	RA, *n* (%)	WA, *n* (%)	RA, *n* (%)	WA, *n* (%)	
M1	22 (57.9)	16 (42.1)	19 (44.2)	24 (55.8)	24 (61.5)	15 (38.5)	25 (71.4)	10 (28.6)	0.104
M2	27 (71.1)	11 (28.9)	23 (53.5)	20 (46.5)	25 (64.1)	14 (35.9)	27 (77.1)	08 (22.9)	0.144
M3	31 (81.6)	07 (18.4)	32 (74.4)	11 (25.6)	34 (87.2)	05 (12.8)	26 (74.3)	09 (25.7)	0.430
M4	14 (36.8)	24 (63.2)	19 (44.2)	24 (55.8)	22 (56.4)	17 (43.6)	17 (48.6)	18 (51.4)	0.374
M5	32 (84.2)	06 (15.8)	39 (90.7)	04 (9.3)	36 (92.3)	03 (7.7)	31 (88.6)	04 (11.4)	0.691
M6	07 (18.4)	31 (81.6)	11 (25.6)	32 (74.4)	12 (30.8)	27 (69.2)	09 (25.7)	26 (74.3)	0.664
M7	28 (73.7)	10 (26.3)	33 (76.7)	10 (23.3)	36 (92.3)	03 (7.7)	26 (74.3)	09 (25.7)	0.141
M8	31 (81.6)	07 (18.4)	34 (79.1)	09 (20.9)	30 (76.9)	09 (23.1)	27 (77.1)	08 (22.9)	0.957
M9	26 (68.4)	12 (31.6)	32 (74.4)	11 (25.6)	27 (69.2)	12 (30.8)	24 (68.6)	11 (31.4)	0.922
M10	21 (55.3)	17 (44.7)	23 (53.5)	20 (46.5)	19 (48.7)	20 (51.3)	20 (57.1)	15 (42.9)	0.898
M11	11 (28.9)	27 (71.1)	29 (67.4)	14 (32.6)	20 (51.3)	19 (48.7)	23 (65.7)	12 (34.3)	0.002^a^
M12	15 (39.5)	23 (60.5)	19 (44.2)	24 (55.8)	14 (35.9)	25 (64.1)	19 (54.3)	16 (45.7)	0.418
M13	25 (65.8)	13 (34.2)	35 (81.4)	08 (18.6)	35 (89.7)	04 (10.3)	27 (77.1)	08 (22.9)	0.077
M14	21 (55.3)	17 (44.7)	28 (65.1)	15 (34.9)	32 (82.1)	07 (17.9)	29 (82.9)	06 (17.1)	0.019^a^
M15	20 (52.6)	18 (47.4)	27 (62.8)	16 (37.2)	28 (71.8)	11 (28.2)	24 (68.6)	11 (31.4)	0.321

M, melody; RA, right answers; WA, wrong answers.

a Statistically significant.

The mean time to perform the test was 3′15″. Reaction time in each song in the total number of children sampled is shown in Table 4.

**Table 4:** Reaction time in each song in total sampled children.

Melody	*n*	Average (SD)	Minimun	Maximun
M1 – *Atirei o pau no gato*	137	11.27 (3.278)	03	25
M2 – *Bate o sino*	145	10.46 (3.732)	03	22
M3 – *Boi da cara preta*	151	9.64 (3.932)	03	38
M4 – *Brilha. brilha estrelinha*	136	11.40 (3.522)	04	26
M5 – *Cai, cai balão*	149	8.64 (3.029)	03	17
M6 – *Capelinha de melão*	132	11.79 (3.952)	02	22
M7 – *Caranguejo*	146	8.75 (3.531)	03	20
M8 – *Escravos de Jó*	143	10.17 (2.886)	03	18
M9 – *Marcha soldado*	147	9.52 (3.348)	03	25
M10 – *Nana nenê*	137	11.36 (3.623)	04	30
M11 – *Noite feliz*	127	10.22 (3.473)	03	23
M12 – *Ó ciranda, ó cirandinha*	131	11.18 (2.924)	05	23
M13 – *O cravo*	149	9.72 (3.405)	02	28
M14 – *Parabéns a você*	143	10.08 (3.495)	03	21
M15 – *Sambalelê*	136	10.26 (3.402)	03	20

SD, standard deviation.

## Discussion

In view of the scarcity of instruments that evaluate the ability of musical recognition, it was observed the necessity of the elaboration of “Evaluation of Recognition of Traditional Melodies in Children”, to assist in the intervention process of children with hearing difficulties (Fig. 1(A–C)).

From the earliest childhood songs to the ever-present popular music of adolescence, music plays an important role in the lives of children. Because of the diffusion of music in all known cultures, children will experience it in a variety of ways daily.^34^

The instrument was performed by means of melodies generated in a piano, according to research carried out^35^ the performances of pianos provided less acoustic clues than the original recordings, however, when the complex signal is simplified and reduced to the melody reproduced in a piano synthesized, the ability to identify music as well as rating, decreases. In this way, the results indicate the positive value that children give to music, even in the face of limited musical information.^36^

According to the results obtained, it was possible to verify that “Cai, cai balão” was the melody most recognized by the children (Table 2), followed by “Boi da cara preta”, “Caranguejo”, “Escravos de Jó”, “O Cravo”, “Parabéns a você” and “Marcha Soldado”, with a recognition percentage superior to 70% of correct. The previous musical experience in children's music education classes may have been a contributing factor to the results obtained. Authors^37^ verified in a study with 5 year-old children, divided into two groups (with or without classes music), that the children enrolled in music classes performed better in a task of musical appreciation in relation to those who had no previous musical knowledge.

Melodies were selected for this study that were part of the musical repertoire observed in the daily life of the children during musicalization classes. It is possible to suggest that during the test application, the children could recognize the melody and not know the title of the same or recognize the melody with a title different from the one proposed in the test. A study^38^ was carried out that obtained the recognition of all students in 27 of the 285 Brazilian folk melodies selected for the study conducted with children from 5 to 12 years of age in the Course of Children Musicalization of the School of Music of the State University of Minas Gerais. The same author observed that during the process of collecting the songs and elaborating the test of recognition of melodies there was similarity between several songs, which in some cases presented identical melody, but different titles; in other cases, the same titles, with different versions. These difficulties found in the folk songbook were an obstacle for the children to identify the songs only by title. Besides the fact that the child could recognize the melody and not know the title of it.

Hypothetically, the observed difficulties^38^ may have been factors that also influenced the process of recognizing melodies of the present study. In another study^39^ with groups of 7 and 11 year-old children, it was found that the increase in the number of errors was inversely proportional to the decrease in reaction time for visual cues presented to children.

Concerning the correlation between the errors and correctness according to age (Table 3), a significant difference was observed only in the melodies “Noite feliz” (*p* < 0.002) and “Parabéns a você” (*p* < 0.019) also revealed that the 9 year-olds had a greater number of hits in relation to the perception of the songs.

The difference between the reception of the stimulus and the time each individual need to initiate a motor response is called “reaction time”.^40^ To determine the speed of motor responses, a measure based on the sum of two components has been used, a central one, called reaction time, and another peripheral, called “time of movement”.^41^ In relation to performance, the lower the reaction time, the greater the efficiency of the central mechanisms and processes.^42^

The reaction time for each melody in all the participants of the research was verified (Table 4), and through the analysis it was concluded that the song “Cai, cai balão” presented the lowest average in relation to the time factor 8.64 s, then came the song “Caranguejo” with 8.75 s, shortly after, “Marcha soldado” with 9.52 s. The song with the longest reaction time on average was “Capelinha de melão” with 11.79 s.

Coincidentally, the songs in which the children showed the shortest reaction time were those with greater recognition by the sample, in this way, it suggests that this result has influence due to the previous knowledge of these songs. This data agrees with reports^43^ that individuality in response time between individuals may be associated with environmental factors and experiences acquired during life.

In addition to being an instrument for the evaluation of sound amplification devices, the Evaluation of Recognition of Traditional Melodies in Children may be used in therapeutic musical training interventions to AID in improvements in neural time, processing speed, auditory work, auditory attention, speech-to-noise comprehension, and auditory scene analysis and cognition.^44^ In this way, research is being conducted with the “Evaluation of Recognition of Traditional Melodies in Children” with different objectives.

## Conclusions

The development and application of the software proved effective for the studied population. This instrument may contribute to the improvement of protocols for the evaluation of musical perception in children with HA and/or CI users.

## Conflicts of interest

The authors declare no conflicts of interest.
